# The Effects of Silibinin Combined With EGFR‐TKIs in the Treatment of NSCLC

**DOI:** 10.1002/cam4.70643

**Published:** 2025-02-05

**Authors:** Xiaocen Wang

**Affiliations:** ^1^ School of Health Medicine University of Sanya Hainan China

**Keywords:** EGFR, nanomedicines, NSCLC, phosphorylation, silibinin

## Abstract

**Background:**

Currently, the most effective oral targeted therapies for NSCLC in clinical practice are EGFR‐TKIs. However, acquired drug resistance often leads to tumor progression and recurrence. EGFR overexpression and activation of its downstream pathways are primary contributors to both mutations in tumor cells and their development of drug resistance. Silibinin has been identified as a promising agent that can suppress EGFR signaling through multiple mechanisms. However, its poor water solubility and difficulty penetrating cell membranes result in rapid metabolism in vivo, and significantly affect its concentration in the blood.

**Methods:**

We conducted a comprehensive search of the English PubMed database using various combinations of keywords, including “silibinin,” “epidermal growth factor receptor,” “phosphorylation,” “chemotherapy,” “nano,” and “non‐small cell lung cancer.” The results were then filtered for their relevance and impact on current treatment paradigms.

**Results:**

This review presents a comprehensive exploration of the mechanisms underlying the EGFR autophosphorylation pathways that contribute to acquire drug resistance in. Additionally, this study delves into the potential of silibinin as a novel therapeutic agent for NSCLC, evaluating its advantages and limitations on the basis of existing research. The majority of the available data suggest that combining silibinin with first‐generation TKIs would yield promising outcomes because of additive or synergistic effects, suggesting that optimizing the time and dosage of each of these treatments is crucial for achieving the best results.

**Conclusion:**

The existing evidence is inadequate to endorse the clinical application of nano silibinin for NSCLC treatment. Developing multifunctional nanomedicines that incorporate silibinin, EGFR‐TKIs, and other bioactive compounds is a recommended future strategy for NSCLC treatment.

## 1 Introduction

Among cancers, lung cancer has the highest rates of incidence and mortality. The latest WHO statistics from 2022 revealed nearly 2,510,000 new cases of lung cancer and 1,800,000 deaths from this disease [1]. The types of lung cancer include non‐small cell lung cancer (NSCLC) and small cell lung cancer (SCLC). NSCLC accounts for approximately 85% of the total cases and is the most common type of lung cancer [2]. Currently, chemotherapy, immunotherapy, radiotherapy, surgery, and targeted therapies are the principal methods which NSCLC is treated. These methods have been widely used in the clinic and have achieved better curative effects. However, the 5 year survival rate of NSCLC patients is 10%–15%. Approximately 40%–55% of NSCLC patients carry epidermal growth factor receptor (EGFR) mutations [3]. The discovery of EGFR tyrosine kinase inhibitors (TKIs) was important in the field of lung cancer treatment. Compared with standard chemotherapy, EGFR‐TKIs have shown superior efficacy in patients with advanced EGFR mutation‐positive NSCLC. EGFR‐TKIs, such as erlotinib, gefitinib, and afatinib, have been approved as first‐line treatments for patients with advanced‐stage EGFR‐mutant NSCLC. However, 20%–30% of these patients exhibit innate resistance and do not respond to initial treatment. Although some patients initially clearly responded to EGFR‐TKIs, 98% of them showed notable response after several months of treatment [4, 5]. Such an imperfect treatment response leads to residual disease, acquired drug resistance in the patient, and is usually fatal. Many mechanisms of innate or acquired drug resistance have been demonstrated in patients. Recent studies have shown that the efficacy of treatment with a single EGFR‐TKI weakens after 3 to 18 months. Thus, it is crucial to improve the efficacy of EGFR‐TKIs against mutated tumor cells to prolong patient survival from the beginning of treatment, and selecting low‐toxicity, high‐efficiency drugs to alleviate resistance to EGFR‐TKIs during the treatment of lung cancer is a solution. *Silybum marianum* (L.) *Gaernt* (Sm)/*Silybum inarianum var. Albiflorum* is known as milk thistle, and has been used as a medicinal material for more than 2000 years [6, 7, 8]. Silibinin is a natural flavonoid derived from milk thistle seeds that has garnered attention for its potential anticancer properties, particularly due to its ability to modulate EGFR signaling [9, 10, 11, 12]. This mechanism of action is pivotal for inhibiting cell proliferation and promoting apoptosis, thereby offering a promising therapeutic strategy against NSCLC.

In this article, the latest progress on understanding the mechanism by which silibinin inhibits cells with EGFR mutations are reviewed. Furthermore, how to increase the effectiveness of EGFR‐TKIs in the treatment of NSCLC is also reported.

## 2 Silibinin Affects the EGFR Pathway

EGFR is the receptor for cell growth and EGF signal transduction [13]. EGFR is located on the surface of the cell membrane and activated upon binding its ligands, including EGF and transforming growth factor α (TGFα). After activation, the EGFR monomer dimerizes. EGFR can also be activated by polymerization with other members of the ErbB receptor family (e.g., ErbB2, HER2, or neu). After dimerization, EGFR can activate kinase pathways in cells via phosphorylation of Y992, Y1045, Y1068, Y1148, and Y1173 [14]. Such autophosphorylation guides the phosphorylation in downstream pathways, including PI3K, RAS, and JAK pathways, and induces cell proliferation. EGFR overexpression plays a key role in the malignant evolution of tumors. EGFR is overexpressed in many tumor cells, such as glioma, renal cell carcinoma, lung cancer, prostate cancer, pancreatic cancer, and breast cancer cells. EGFR is related to the inhibition of tumor cell proliferation, angiogenesis, tumor invasion, metastasis and apoptosis. Therefore, EGFR is regarded as a target for anticancer medicines [15]. By examining how silibinin affects EGFR‐mediated pathways, we can further elucidate the comprehensive mechanisms by which silibinin exerts its antitumor effects, paving the way for more targeted and effective cancer treatments.

### 2.1 Silibinin Inhibits the PI3K‐AKT–mTOR Pathway

PI3K‐AKT–mTOR is one of the classical signal transduction pathways that has been proven to play an important role in the development of cancer. Mammalian target of rapamycin (mTOR) is an important kinase downstream of PI3K‐AKT that regulates cancer cell angiogenesis, growth, proliferation and survival. Cancer cells escape the normal biochemical system that regulates the balance between apoptosis and survival. PI3K‐AKT–mTOR usually stimulates apoptosis‐promoting factors and activates apoptosis inhibitors. Upon phosphorylation, PI3K‐AKT–mTOR signaling suppresses the activity of apoptosis‐promoting factors whereas activating factors that prevent apoptosis. The phosphatase PTEN negatively regulates PI3K. A decrease in PTEN expression indirectly supresses PI3K‐AKT–mTOR signaling and promotes tumor development. According to recent data, the self‐renewal properties of cancer stem cells and resistance to chemotherapy or radiotherapy are closely related to PI3K‐AKT–mTOR signaling. This pathway is considered the causes of treatment failure, cancer recurrence and metastasis.

Research has shown clarified that silibinin can inhibit the PI3K‐AKT–mTOR pathway in multiple cancer cell models. Chen et al. utilized A549 cells to study the effects of silibinin on the PI3K‐AKT signaling pathway and reported that AKT phosphorylation was inhibited by 63.3% upon treatment with 100 μmol/L silibinin. This result directly supports the inhibitory efficacy of silibinin in lung cancer. The growth of bladder cancer cells was inhibited by silibinin in a dose dependent manner. T24 and UM‐UC‐3 cells were treated with 5, 10 or 25 μmol/L silibinin. A cell proliferation assay revealed that silibinin delayed T24 cell proliferation by 20% (5 μmol/L), 69% (10 μmol/L), and 93% (25 μmol/L) after 48 h. Silibinin also suppressed UM‐UC‐3 cell proliferation by 39% (5 μmol/L), 69% (10 μmol/L), and 77% (25 μmol/L) after 48 h [16]. Silibinin significantly suppresses the RAS‐driven actin cytoskeleton and PI3K‐AKT pathways significantly [17]. Lin et al. reported that pretreating retinal pigmented epithelial (RPE) cells were pretreated with 100 μmol/L silibinin, inhibited the phsophorylation of AKT, mTOR and p70 S6K. Ribosomal protein S6 kinase1 (S6K1) is an important downstream effector of mTOR that regulates and mediates many metabolic diseases including obesity, type 2 diabetes and cancer. Thus, S6K1 has become an important therapeutic target for these diseases [18]. Lin et al. [19] showed that silibinin had blocked the phosphorylation of S6K1 in MCF‐7 cells. Li et al. [20] used 50 μmol/L silibinin to treat renal cell carcinoma (RCC) cells for 24 h. They reported that silibinin suppressed the phosphorylation of mTOR at Ser‐2448 and S6K at Ser‐371 and Thr‐389. These studies also revealed that silibinin suppressed the PI3K‐AKT–mTOR signaling in a dose‐dependent manner. It is important that a sufficient the enough concentration of silibinin reaches the targeted cells in order to achieve better curative effects.

### 2.2 Silibinin Inhibits the RAS–RAF–MEK–ERK Pathway

On the basis of in‐depth studies from the last few decades, RAS–RAF–MEK–ERK has been considered a classic tumor signaling pathway. This signaling pathway is closely related to the development of many cancers. First, RAS proteins with GTPase activity are activated by upstream RTKs. Once activated, RAS activates RAF by binding to the N‐terminal domain of RAF and activated RAF binds to and activates the downstream protein MEK. MEK activates the downstream substrate ERK only, and finally, activated ERK enters the cell nucleus to trigger a series of physiological and biochemical reactions. Although mutations in MEK1/2 are not as common as B‐Raf and RAS mutations are, MEK1/2 are ideal therapeutic targets because of they are downstream of RAS and RAF, which have relatively high mutation rates, approximately 25% of these cause activation of proteins in the RAS family [21].

MCF‐7 breast cancer cells were used to research the effects of silibinin on MMP‐9 and VEGF expression after TPA induction. The authors reported that phosphorylation of TPA‐induced RAF, MEK, and ERK decreased 30 min after the administration of by 100 μmol/L silibinin [22]. MCF‐7 and MDA‐MB‐231 cells were used to investigate silibinin‐induced cell death and suppression of the ERK signaling pathway [23]. Chen et al. [16] reported that silibinin inhibited ERK and AKT phosphorylation in A549 cells, which restricted activation of the PI3K‐AKT and MAPK signaling pathways. Thus, silibinin has the to limit the proliferations of lung cancer cells.

### 2.3 Silibinin Inhibits the IL‐6R/JAK/STAT3 Pathway

The gene encoding STAT3 is located on chromosome 17q21 and 24 exons. The gene is 4815 bp in length, and the mature protein adopts a knot configuration and is composed of 750–800 amino acids. Synthetic proteins are divided into three subtypes: *α*, *β*, and *γ* [24]. STAT3 is a key signaling molecule that affects inflammation in tumors. The activation of STAT3 involves two main mechanisms: nuclear activation via Tyr705 phosphorylation and mitochondrial activation via Ser727 phosphorylation. STAT3 is transiently activated upon phosphorylation, which allows the transmission of transcriptional signals from cytokine and growth factor receptors on the plasma membrane to the nucleus. Once activated, STAT3 primarily promotes tumor cell growth, metastasis, and immune evasion. In the tumor microenvironment, the inflammatory factor interleukin‐6 (IL‐6) signals through the IL‐6R/ JAK/ STAT3 pathway. This pathway regulates the genesis and development of tumor cells. Previous studies have shown that IL‐6 binds to GP130 to form the GP130/IL‐6 receptor complex. After the receptor‐related residue family of kinase, Janus family tyrosine kinases (JAKs), are activated by GP130/IL‐6 via cross‐phosphorylation, the activated JAKs phosphorylate the tyrosine residues in the receptor molecule, which allows cytoplasmic STAT3. Then a single tyrosine residue of STAT3 is phosphorlated (p‐STAT3) [25]. P‐STAT3 first forms a homodimer in the cytoplasm, which is translocated to the nucleus, where it binds the target DNA sequence and regulates the expression of target genes [26, 27]. Moreover, the JAK/STAT3 pathway is one of the main pathways downstream of EGFR [28]. Once it is expressed, STAT3 transmits information to tumor cells and acts on its target genes to regulate tumor cell proliferation, differentiation and metastasis [29].

P‐STAT3 was shown to be inhibited by silibinin in a dose‐dependent manner. Tumor‐bearing model mice are often used to examine the ability of silibinin to inhibit STAT3 phosphorylation. An in vitro cell screening assay was used to investigate the effects of silibinin on the inhibition of p‐STAT3. In an inbred albino strain (A/J) mouse model, silibinin was shown to have antiangiogenic effects on established lung adenocarcinomas and inhibit their growth and progression. According to the report of Tyagi et al., silibinin strongly reduced both p‐STAT3 (Ser727) activation in tumor cells and the population of lung macrophages. In multiple pulmonary tumors induced by urethane in wild‐type (WT) model mice, silibinin significantly suppressed the activities of STAT3 and NF‐ĸB. There was a 38% reduction in the number of nuclear p‐STAT3 (Ser727)‐positive cells after silibinin treatment compared with after control treatment. Moreover, compared with that in the control group, the number of nuclear p65NF‐ĸB (Ser276)‐positive cells decreased by 31% after silibinin treatment [30]. These results revealed that silibinin inhibited the expression of STAT3 and NF‐ĸB in pulmonary tumor cells. STAT3 and NF‐ĸB bind to the promoter region of the iNOS gene. This downregulation of iNOS is likely the mechanism of the chemopreventive and vascular preventive effects of silibinin [31]. Serum‐starved A549 cells were treated with different doses of silibinin (50 to 200 μmol/L) for 24 h. After incubation, silibinin was found to significantly block the phosphorylation both uninduced and cytokine mixture‐induced STAT3 at Tyr705. Silibinin also inhibited STAT3 phosphorylation at Ser727. Silibinin treatment can reduce the total STAT3 level after induction with mixture of cytokine [32]. Bosch‐Barrera and Menendez predicted that silibinin could reduce the inducibility, formation and acquired STAT3 feedback due to activation at the Tyr705 site. Silibinin attenuated Try705‐induced phosphorylation of the green fluorescent protein (GFP)‐STAT3 gene fusion protein [33]. However, the kinase activities of JAK1 and JAK2 were not significantly changed. In silico studies revealed that silibinin could bind directly to both the SH2 domain and the DNA‐binding domain (DBD) of STAT3 with high affinity. Because silibinin occupied the space in the SH2 domain that was previously used by STAT3, silibinin was able to indirectly prevent the phosphorylation of Tyr705 phosphorylation indirectly. Furthermore, silibinin was predicted to disturb the binding between the STAT3 DBD and DNA. As an inhibitor of STAT3, silibinin prevents STAT3 nuclear translocation, blocks its binding to DNA that would activate STAT3 and suppresses the transcriptional activity of STAT3 [34]. Furthermore, silibinin might inhibit both the constitutive and inducible activities of STAT3, and reduce the expression of the proteins encoded by the STAT3 target genes, such as Cyclin D1 and JAK2 [30].

### 2.4 Silibinin Inhibits NF‐ĸB

Nuclear factor activated light chain enhancement in B cells (NF‐κB) is a protein complex that controls DNA transcription, cytokine production and cell survival. In almost all animal cells, NF‐κB participates in cellular responses to stimuli, such as stress, cytokines, free radicals, heavy metals, UV irradiation, oxidized LDL, and bacterial or viral antigens. NF‐κB also plays a crucial role in regulating the immune response to infection. Dysregulation of NF‐κB expression is associated with cancer, inflammation, autoimmune diseases, septic shock, viral infections, and inadequate development of the immune system. NF‐κB is activated in tumor cells because of mutations in genes encoding the NF‐κB transcription factors themselves or genes that control NF‐κB activity. Blocking NF‐κB causes tumor cells to stop proliferating, die or become more sensitive to the effects of antineoplastic agents. Therefore, NF‐κB has generally been studied as a target for anticancer therapy. NF‐ĸB is a downstream effector of EGFR. Since the overexpression of EGFR can be detected, inhibiting of NF‐ĸB leads to the downregulation of EGFR in cells [35]. Silibinin suppresses the EGFR signaling pathway by blocking downstream signal transduction, and regulating of other related signaling pathways. Silibinin is considered a potential drug for treating cancer metastasis, including lung cancer metastasis [36]. The effects of silibinin on EGFR pathway suppression are presented in Table 1.

**TABLE 1:** Effects of silibinin on EGFR pathway suppression.

Cancer type	Cell models	Silibinin dose (μmol/L)	Incubation duration (h)	Outcome and *p*	Suppressed pathway	Ref.
Bladder cancer	T24	0 (control)	48	Decreased cell proliferation	PI3K‐AKT–mTOR	Imai‐Sumida [17]
		5		*		
		10		***		
		25		***		
Bladder cancer	UM‐UC‐3	0 (control)	48	Decreased cell proliferation	PI3K‐AKT–mTOR	Imai‐Sumida [17]
		5		**		
		10		***		
		25		***		
Breast cancer	MCF‐7	0 (control)	24	Decreased cell proliferation	PI3K‐AKT–mTOR	Lin [19]
		50		**		
		100		**		
Breast cancer	MCF‐7	0 (control)	48	Inhibition of phosphorylation	RAS–RAF–MEK–ERK	Kim [22]
		10		Null		
		25		*		
		50		*		
		100		**		
Breast cancer	MDA‐MB‐231	0 (control)	48	Inhibition of phosphorylation	RAS–RAF–MEK–ERK	Kim [23]
		5		Null		
		10		*		
		20		*		
		30		**		
		50		**		
Lung cancer	A549	0 (control)	24	Inhibition of phosphorylation	PI3K‐Akt–mTOR// RAS–RAF–MEK–ERK//NF‐kB	Chen [16]
		30		Null/null/**		
		50		Null/**/**		
		70		Null/**/***		
		100		**/***/***		
Lung cancer	A549	0 (control)	24	Inhibition of phosphorylation	STAT3	Chittezhath [32]
		50		*		
		100		*		
		200		*		

* *p* < 0.05.

** *p* < 0.01.

*** *p* < 0.001.

## 3 Silibinin Combined With EGFR‐TKIs in the Treatment of NSCLC

**FIGURE 1:**
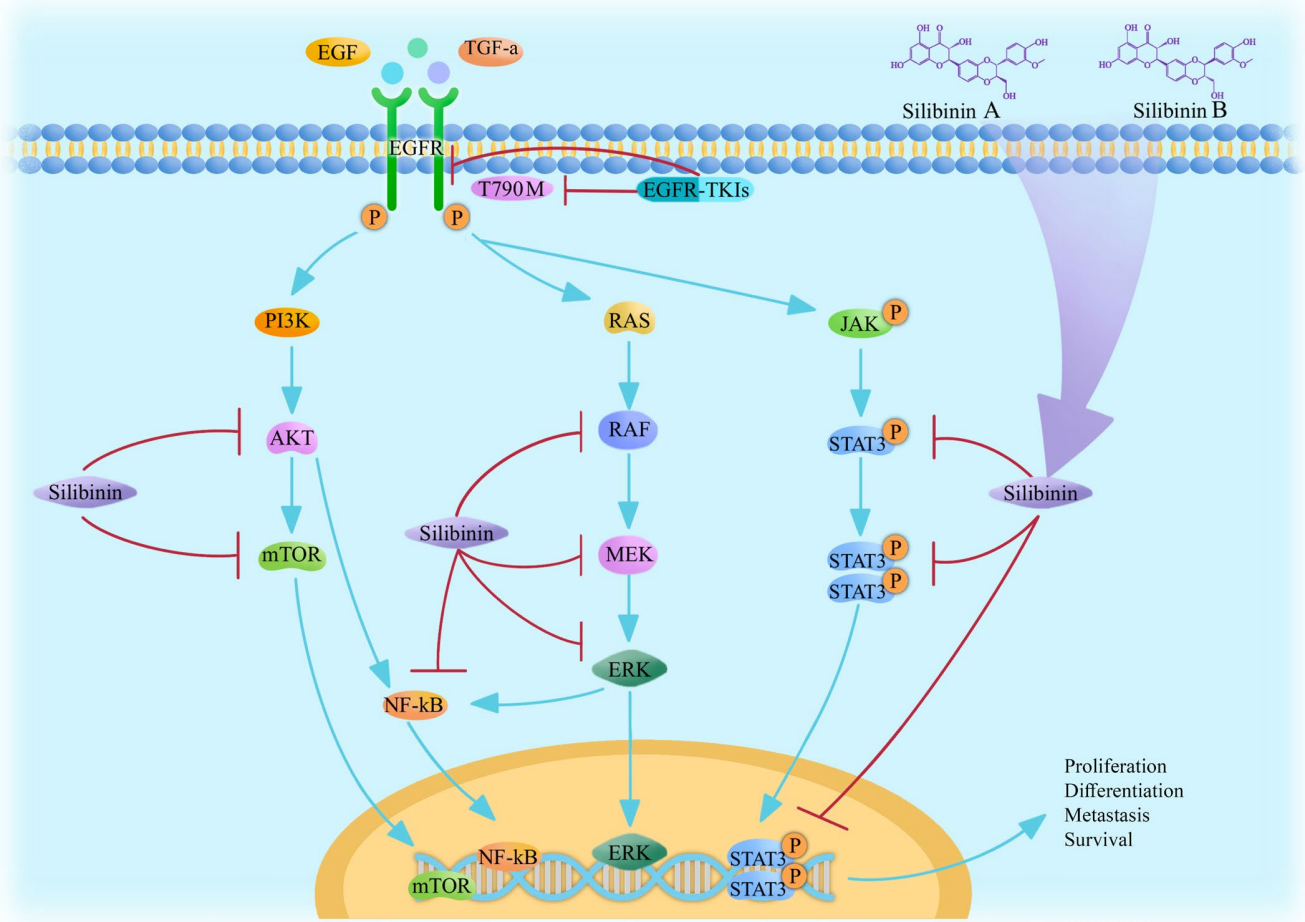
EGFR‐TKIs and silibinin suppress the downregulation of EGFR in NSCLC cells.

EGFR is a glycoprotein and a tyrosine kinase receptor. EGFR can penetrate the cell membrane and has a molecular weight of 170 kDa [37]. Although EGFR‐TKIs are highly effective in the treatment of lung cancer, drug resistance can develop after drug administration for a period duration. Most patients are resistant to targeted drugs after 3 to 18 months of treatment, and few patients can take the same drug for 3–4 years. At present, the main mechanism of acquired drug resistance to EGFR‐TKIs is secondary mutations in factors of the EGFR pathway. Approximately 50% of the patients that were treated with first‐generation EGFR‐TKIs acquired drug resistance through secondary mutation to exon 20, producing threonine 790 mutant cells (T790M). Other mechanisms acquired drug resistance include activation of alternative or downstream pathways, such as human encoded MET protein (c‐MET) amplification, HER‐2 mutations, phenotypic changes and tumor heterogeneity [38]. Silibinin is considered an inhibitor of EGFR phosphorylation. To a certain extent, silibinin can inhibit NSCLC growth and metastasis. EGFR‐TKIs generally inhibit EGFR shortly after the mutation occurs, but acquired drug resistance usually occurs during treatment. However, silibinin can counteract EGFR mutations by inhibiting the factors in the secondary pathways that are phosphorylated by EGFR. Therefore, silibinin delays resistance. The pathways by which EGFR‐TKIs and silibinin suppress the downregulation of EGFR in NSCLC cells are shown in Figure 1.

### 3.1 Combining Silibinin With EGFR‐TKIs In Vitro

The secondary mutation T790M is related to approximately 50% of acquired drug resistance. The third‐generation EGFR‐TKI, such as osimertinib, can irreversibly bind to the EGFR receptor. This drug has a more compact molecular structure, which enhances its efficacy against the T790M mutation. The acquired resistance typically emerges after an average of 18 months of treatment with osimertinib, a phenomenon attributed to the activation of EGFR downstream signaling pathways. Silibinin is thought to selectively reduce the activity of EGFR family members (EGFR, ErbB2 and ErbB3) by inhibiting the dimerization of mutated EGFR in lung cancer cells. In primary and drug‐resistant cells with the T790M mutation, the inhibitory effect of the EGFR‐TKI on EGFR signaling was significantly enhanced after silibinin administration. The anaplastic lymphoma kinase (ALK) gene is presented in 2%–5% of NSCLCs. ALK is activated via the PI3K/ AKT, ERK1/2/MAPK and STAT3 pathways and is thus often researched together with EGFR. Crizotinib is an oral tyrosine kinase receptor inhibitor. This drug is used to treat patients who suffer from advanced NSCLC and abnormally express ALK. EGFR has been found to be hyperactivated in crizotinib‐resistant H3122/CR cells [39]. Cuyàs et al. researched the effects of silibinin on crizotinib resistance in ALK‐translocated NSCLC cells. They reported that combining crizotinib with silibinin significantly improved EGFR hyperactivation in H3122/CR cells due to the silibinin‐mediated inhibition of STAT3 activation [40].

Erlotinib combined with silibinin significantly inhibited tumor growth. Silibinin significantly reversed the epithelial mesenchymal transition (EMT) mediated high expression of miR‐21 or low expression of miR‐200c and inhibited the mesenchymal markers snail, ZEB and N‐cadherin in erlotinib‐induced intractable tumors [41]. Compared with that of erlotinib alone, the proliferation of PC‐9/ER cells was reduced by 200% in the presence of both 100 μmol/L silibinin and 1 μmol/L erlotinib [42]. In erlotinib‐resistant cells, the expression of the aldehyde dehydrogenase (ALDH) gene was more than 45 times greater that in erlotinib‐responsive cells. After incubation with 100 μg/mL silibinin‐meglumine, the proportion of ALDH cells resistant to erlotinib decreased by 80%. Moreover, in cancer stem cells (CSCs), silibinin works with erlotinib to eliminate the formation of tumors in a dose‐dependent manner [43]. These results clearly emphasize the benefits of using silibinin in combination with erlotinib, providing targets in CSCs and minimizing the possibility of tumor cell survival in NSCLC patients with EGFR mutations.

Cells from nonsmoking female NSCLC patients (H1975) and gefitinib‐resistant lung cancer cells (PC‐9/GR) can autocrinally produce IL‐6, activate the IL‐6R/JAK/STAT3 signaling pathway, and increase the activity of STAT3, which lead to mutations. Cufi reported that silibinin combined with gefitinib inhibited the regrowth of gefitinib‐resistant tumor cells. Additionally, EMT was impeded by silibinin administration. Compared with gefitinib alone, 100 μmol/L silibinin plus 1 μmol/L gefitinib reduced the proliferation of H1975 cells by 50%. Furthermore, compared with that after treatment with gefitinib alone, the PC‐9/GR cell proliferation was reduced by 163% after combined treatment with the 100 μmol/L silibinin and 1 μmol/L gefitinib [41]. Nintedanib is a triple vascular kinase inhibitor that can simultaneously block signal transduction by vascular endothelial growth factor receptor (VEGFR), platelet‐derived growth factor receptor (PDGFR) and fibroblast growth factor receptor (FGFR). Nintedanib is usually used to treat NSCLC after first‐line chemotherapy has been attempted. Bosch‐Barrera et al. researched how silibinin affects nintedanib treatment and reported that silibinin combined with nintedanib significantly inhibited the viabililty of the NSCLC cell lines A549, H460, H1933, H1975, H2228, H3122, and PC9. Furthermore, the EGFR mutants were suppressed in these models. They also reported that silibinin reduced the content of nintedanib in the lysosomes that occurs in nintedanib‐resistant NSCLC cells and increased the ability of nintedanib to reach its intracellular targets [44]. The results of combining silibinin with other chemotherapy drugs for NSCLC treatment in vitro are presented in Table 2.

**TABLE 2:** In vitro effects of silibinin combined with other chemotherapy drugs for NSCLC treatment.

Combination treatment	Cell models	Silibinin dose	Treatment duration	Outcome and *p*	Ref.
Crizotinib	H3122/CR	0 (control)	24 h	The concentration of drug required to reduce cell viability by 50% (IC50)	Cuyas [40]
		50 μmol/L		**	
Erlotinib	PC‐9/ER	0 (control)	72 h	Decreased number of cells	Rho [42]
		100 μmol/L		**	
Gefitinib	PC‐9/GR	0 (control)	72 h	Decreased number cells	Rho [42]
		100 μmol/L		**	
Erlotinib	PC‐9/ER	0 (control)	72 h	The proportion of ALDH cells resistant to erlotinib decreased.	Corominas‐Faja [43]
		100 μg/mL		**	
Nintedanib	A549 H460 H1993 H3122 H2228 PC9	0 (control)	10 days	The concentration of drug required to reduce cell viability by 50% (IC50)	Bosch‐Barrera [44]
		100 μmol/L		**	

** *p* < 0.01.

In vitro experiments, silibinin significantly inhibited the proliferation of drug‐resistant lung cancer cells because the EGFR‐TKIs, silibinin and cells were prepared under controlled conditions. However, in vivo, drug efficacy is affected by its absorption, distribution, metabolism, and excretion. To elucidate the synergistic effects of silibinin combined with EGFR‐TKIs, scientists have conducted some in vivo studies.

### 3.2 Silibinin Plus EGFR‐TKIs Combined Treatment In Vivo

Silibinin has poor aqueous solubility, so it is usually prepared as a meglumine salt to increase its solubility. Cufi et al. compared the effects of gefitinib and silibinin meglumine + gefitinib in the treatment of NSCLC. Compared with gefitinib treatment alone, the suppressive effect of silibinin combined with gefitinib displayed an increase of 22.02% [45]. The combination of silibinin and EGFR‐TKIs is considered a promising strategy to overcome T790M‐mediated drug resistance [41, 42]. The combination of silibinin and erlotinib significantly inhibited the growth of erlotinib‐resistant PC‐9 xenografts tumors. PC‐9/ER tumor cell xenografts were treated with placebo, erlotinib (100 mg/kg), silibinin (200 mg/kg) or silibinin (200 mg/kg) plus erlotinib (100 mg/kg) for 24 days. Although the tumor cells continued to proliferate, silibinin+erlotinib treatment significantly decreased the speed of proliferation. These promising results are shown in Table 3.

**TABLE 3:** Tumor volumes after treatment with a combination of silibinin plus another anticancer therapy in vivo.

Animal models	Treatment duration	Control	Combination treatment drug			Ref.
PC‐9 xenograft	35 days	Placebo	Gefitinib	Silibinin	Silibinin + Gefitinib	Cufi [45]
Tumor volume (mm^3^)		327 ± 45	168 ± 40*	151 ± 39*	96 ± 14**	
PC‐9/ER xenograft	35 days	Placebo	Erlotinib	Silibinin	Silibnin + Erlotinib	Cufi [41]
Tumor volume (mm^3^)		953 ± 90	762 ± 80	452 ± 45*	134 ± 16**	

* *p* < 0.05.

** *p* < 0.01.

**FIGURE 2:**
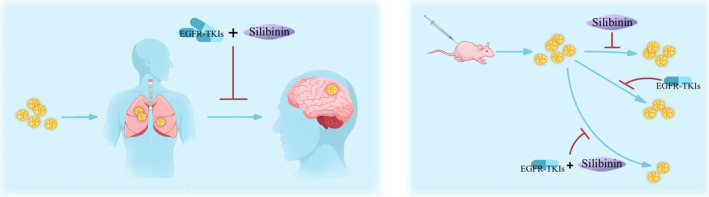
Combining EGFR‐TKIs with silibinin showed promising tumor inhibition effects in both animal models and patients [41, 46].

Previous findings confirmed that silibinin could pass through the blood brain barrier (BBB) and suppress STAT3 activation. Therefore, it has been used to treat the brain metastasis of lung cancer. The patients who received both Legasil and EGFR‐TKI therapy had a median survival of 15.5 months (95% CI: 9.4–21.7), whereas the patients who were treated with only EGFR‐TKI therapy had a median survival of 4.0 months (95% CI: 3.2–4.9), *p* < 0.01 [46]. An illustration depicting this type of therapy is shown in Figure 2.

## 4 Application of a Silibinin Nanocarrier in the Treatment of NSCLC

**FIGURE 3:**
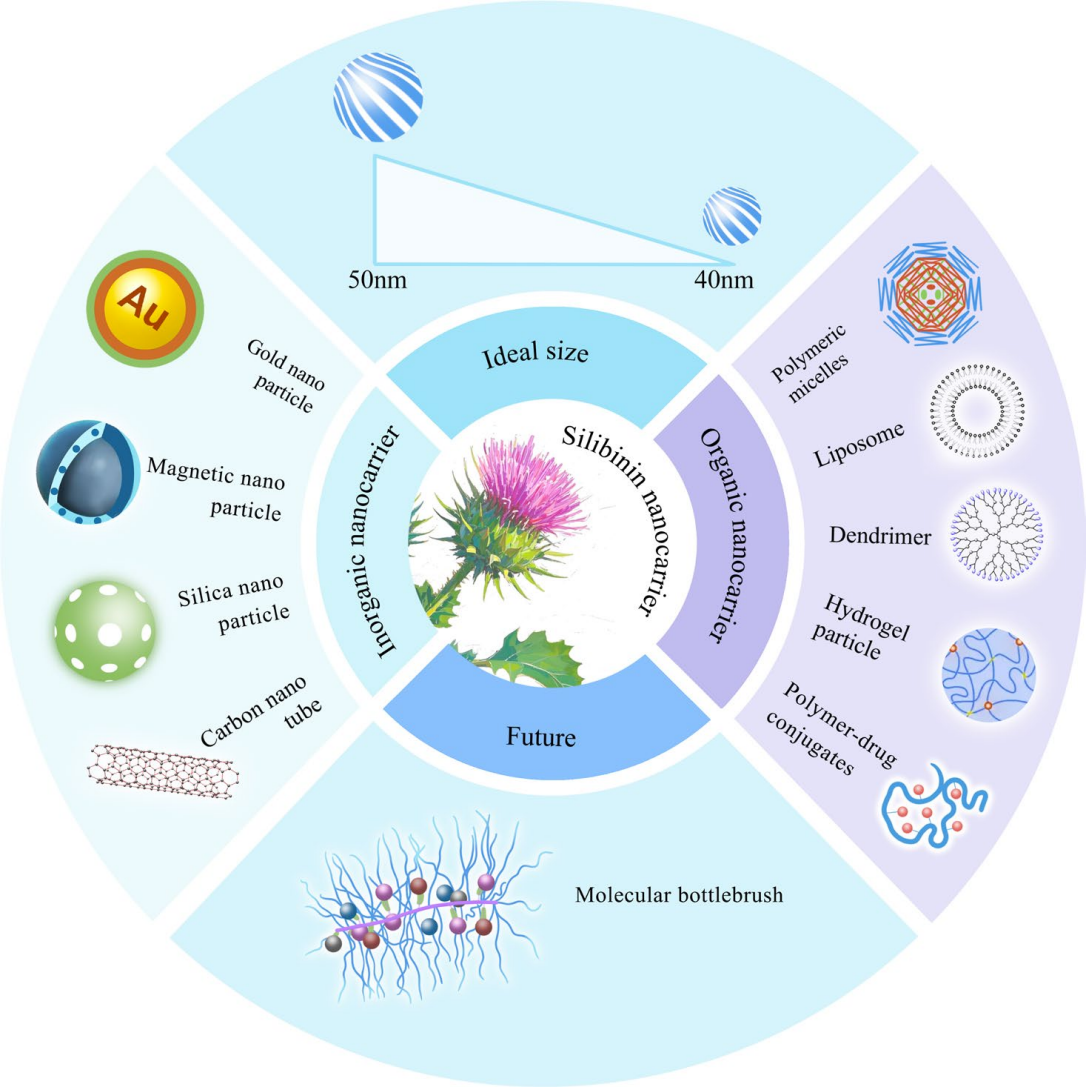
The main forms and ideal states of silibinin nanocarriers.

The low aqueous solubility, weak penetration, high metabolism and rapid elimination of silibinin limit its efficacy and clinical applications. Nanodrug delivery systems (NDDSs) can enhance drug solubility, modify distribution of the drug in the body, and increase drug targeting, thereby improving therapeutic effects of the drug whereas reducing adverse reactions. NDDSs have emerged as a focal point in targeted cancer therapy research. Previous studies have shown that silibinin, when carried by various nanocarriers, including metal, silica, and carbon nanomaterials, polymeric micelles, liposomes, dendritic molecular carriers, hydrogels, and polymer‐drug conjugates, exhibits superior pharmacokinetic properties in both in vivo and in vitro for the treatment of NSCLC and other cancers than silibinin delivered via conventional methods [47, 48, 49, 50]. Silibinin nanocarriers are shown in Figure 3.

Mogheri et al. coloaded metformin and silibinin in polymeric nanoparticles (NPs) fabricated from poly (D,L‐lactide‐co‐glycolide)‐polyethylene glycol (PLGA‐PEG). They investigated the combined inhibitory effects of these compounds on A549 human lung cancer cells and determined IC50 values for metformin‐NPs, silibinin‐NPs, and dual‐drug‐loaded NPs of 9.75 mmol/L, 36.11 μmol/L, and 0.85 μmol/L, respectively. Notably, these values were significantly lower than those of their pure counterparts. Additionally, the study demonstrated that silibinin‐NPs exhibited a considerably greater cancer cell elimination effect than did the metformin‐NPs. Overall, Mogheri's highlighted that dual‐drug‐loaded NPs exerted substantial synergistic toxicity to A549 lung carcinoma cells, surpassing the efficacy of either single drug formulation [51]. Salmani‐Javan et al. utilized niosomal‐PEG technology to develop a drug nanocarrier system and assessed the effects of metformin‐NPs and silibinin‐NPs on A549 lung cancer cells. The study revealed that the IC50 for the metformin‐NPs was 68.42 μmol/L, whereas that for the silibinin‐NPs was significantly lower at 10.36 μmol/L. This comparison underscores the enhanced efficacy of silibinin encapsulated in a nanoparticle formulation compared with encapsulated metformin [52]. Chitosan‐based delivery systems have shown promise for silibinin delivery, by enhancing its antitumor effects. Kuen et al. evaluated the efficacy of hydrophobically‐modified chitosan nanoparticles as carriers for silibinin in lung cancer therapy. Their findings revealed that encapsulating silibinin within these nanoparticles significantly improved both its dissolution rate and cytotoxicity compared with free silibinin [53]. Patel et al. also discovered that, under identical incubation conditions, the concentration of folic acid‐chitosan‐silibinin‐NPs remained higher in lung tissues than the concentration of chitosan‐silibinin‐NPs [54]. Raval et al. demonstrated that silibinin was released more slowly from chitosan‐NPs than was free silibinin in vitro. Additionally, compared with the other formulations, the chitosan‐PLGA‐NPs significantly inhibited A549 cells. The optimized silibinin‐NPs were further adapted for inhalation administration, with a fine particle fraction of 80.2%, indicating their suitability for effective pulmonary administration [55]. These NPs penetrated deep into lung tissue, which promoted the cellular adhesion and retention of the delivery system, thus enabling both local and systemic effects. The NPs also maintained the drug concentration in the lungs for a longer duration. An in vivo pharmacokinetic study in rats strongly indicated that both the rate and extent of silibinin bioavailability was markedly improved upon its encapsulation in chitosan‐PLGA‐NPs. Consequently, recent efforts have focused on enhancing the deposition of NPs in lung tumors and reducing their clearance to improve their efficacy and control.

A previous study showed the potential of using PLGA‐PEG‐Fe_3_O_4_ nanoparticles, as effective targeted carriers to target lung cancer cells [56]. Furthermore, compared with that in normal tissues, significantly more silibinin‐NPs accumulated in cancer tissues. Additionally, gold‐silibinin nanoconjugates displayed enhanced cytotoxic and growth inhibitory effects compared with silibinin alone [57]. Compared with free silibinin, silibinin‐MWCNTs exhibited enhanced toxicity to human cancer cell lines at lower concentrations [58]. These results are shown in Table 4.

**TABLE 4:** Silibinin nanocarriers in lung cancer treatment.

Phytochemical	NDDSs	Combination drug/ligand/conjugate	Substance size (nm)	Entrapment efficiency (EE%)	Loading capacity (LC%)	Cell line/animal model	Findings compared with the native drug and *p*	Ref.
Silibinin	PLGA‐PEG NPs	Metformin	223 ± 9.43	67.4 ± 3.5	13.6 ± 3.1	A549	Cytotoxicity↑*** Apoptosis↑***	Mogheri [51]
	PEGylated‐niosomeal NPs	Metformin	162.5 ± 1.8	—	—	A549	Cytotoxicity↑*** Apoptosis↑***	Salmani‐Javan [52]
	Chitosan NPs	—	208 ± 12.02	49.33 ± 1.45	—		Cytotoxicity↑*	Kuen [53]
	Chitosan NPs	Folic acid	275 ± 1.20	75.52 ± 0.87	—	A549 cells Sprague–Dawley rat	In vitro cytotoxicity↑* In vivo tumor volume/weight↓*	Patel [54]
	Chitosan‐PLGA NPs	—	230–284	56.8–65.5	35.1–45.4	A549 cells Sprague–Dawley rats	In vitro cytotoxicity↑* In vivo drug concentration, mean residence time and half‐life↑*	Raval [55]
	Magnetic NPs	—	130–150	—	—	A549	Cytotoxicity↑*	Amirsaadat [56]
	Au NPs	—	200–900	—	—	A549	Cytotoxicity↑ Apoptosis↑	Ravi [57]
	Carbon NTs	COOH‐MWCNTs	Outside diameter 20–30, length 0.5–2.0 μm	—	—	A549	Cytotoxicity↑* at lower concentrations, cytotoxicity↓* at higher concentrations	Tan [58]

*Note:* ↑‐enhanced, ↓‐reduced.

* *p* < 0.05.

*** *p* < 0.001.

In addition to silibinin, other compounds such as atractylenolide III, baicalin, betulinic acid, chrysotoxine, dieckol, eupatolide, guttiferone E, honokiol, luteolin, parthenolide, and resveratrol are known to target EGFR signaling and inhibit the proliferation of NSCLC cancer cells with substantial efficacy. In future studies, these natural compounds could be used alone or in combination with other therapies to provide novel strategies for treating NSCLC [59, 60, 61, 62, 63, 64, 65, 66, 67, 68, 69, 70, 71, 72, 73].

## 5 Discussion

EGFR is expressed on the surface of all cells and plays important roles in controlling cell growth, death and other functions. When EGFR is mutated, cell growth becomes uncontrolled, and the cells become a tumor. In NSCLC, the frequency of EGFR gene mutations is very high, especially in individual of Asian descent and nonsmoking women, with an incidence of more than 50%. However, the mutation rate of the EGFR lung cancer patients in Europe and America is only approximately 15%. Signaling pathway blockers are used to terminate signal transmission to weaken the EGFR mutant. EGFR‐TKIs can competitively block the binding of ATP to mutant EGFR, to preventing the transmission of growth signals, and starving tumor cells, ultimately inhibiting the cancer cells. However, acquired drug resistance to EGFR‐TKIs is inevitable. The relationship between the potential effects of silibinin and the reversal of resistance to TKIs through the inhibition of secondary pathways has been studied in EGFR‐mutated tumors. According to previous research, silibinin not only blocks signal transduction by inhibiting the phosphorylation of signaling factors downstream of EGFR, but also improves the effects of EGFR‐TKIs in vivo and in vitro. Previous experimental research revealed that silibinin could overcome resistance to gefitinib and erlotinib both in vitro and in vivo.

Although the concentrations of silibinin can be relatively high in vitro, the relatively lower concentrations of silibinin have been found in vivo. In previous studies, maintaining the concentration of silibinin in human blood was challenging, which greatly hindered its application as a preventive cancer medicine or the anticancer drug. Silibinin has several limitations because of its poor aqueous solubility, low intestinal epithelial cells permeability, extensive metabolism and rapid systemic clearance, which limit its bioavailability in vivo.

The main advantages of nanotechnology‐based drugs and drug delivery systems are their effective targeting, and sustained drug release properties, prolonged half‐life and reduced systemic toxicity. Compared with traditional methods, the use of nanodrugs has significantly improved the delivery of drugs to their targets. It has previously been shown that nanocarriers can effectively increase the plasma concentration of silibinin and overcome the problem of its poor bioavailability. Such nanocarriers can transport hydrophobic and hydrophilic molecules, exhibit extremely low or no toxicity, extend the duration of drug delivery and enable controlled drug release for a longer half‐life.

Nanocarrier‐encapsulated drugs differ structurally from those not in nanocarriers. Consequently, when assessing the active drug concentration in systemic circulation and the in vivo absorption characteristics, it is essential to consider the concentration of the drug carrier in the blood. Many inorganic metal nanoparticles can cause DNA damage, oxidative stress responses, and cell damage. Organic nanoparticles are relatively safe but may polymerize and become toxic. Therefore, the safety and metabolism of nanocarrier drugs must be evaluated before their use. Additionally, the particle size of the nanocarrier directly affects it's in vivo absorption. Larger nanoparticles entering the bloodstream can be recognized and cleared by proteins in the blood, which makes the size of a nanomedicine an important consideration. Nanoparticles less than 100 nm in size can effectively reside within solid tumors, although their surfaces need to be hydrophilic to achieve long‐circulation times in the bloodstream, and increase their accumulation. Studies have shown that if nanoparticles are too large, cells struggle to internalize them and they remain in the body for a long time; conversely, if they are too small, they cannot bind to membrane surface receptors and are quickly cleared via the urine. The optimal size for nanodrug carriers is between 40 and 50 nm [74]. In previous experiments, most silibinin‐NPs exceeded 100 nm. For safety and efficacy considerations, nanoparticles of this size are not suitable for clinical trials.

Compared with the use of silibinin alone, combining silibinin with other drugs and its encapsulation in a nanocarrier significantly enhances its ability to inhibit tumor cell growth and signaling. When designing nanocarriers, factors such as the type of carrier, the rigidity and flexibility of the nanoparticles, and cell selectivity are crucial. Therefore, creating efficient, safe, and smart targeted nanodrug delivery systems and thoroughly understanding their behavior in the body are essential for advancing nanodrug delivery technologies.

## 6 Conclusion

In cases where first‐ and second‐generation EGFR‐TKIs fail to inhibit the binding of ATP to the EGFR tyrosine kinase active site, and where third‐generation EGFR‐TKIs inadequately inhibit T790M‐mutation EGFR, silibinin could effectively suppress tumor initiation and development by blocking downstream signaling pathways in tumor cells [75]. Both in vitro and in vivo experiments have demonstrated that when combined with EGFR‐TKIs and other anticancer drugs, silibinin has excellent synergistic effects. Compared with traditional drug delivery methods, nanocarriers significantly increase the solubility, duration of action, and inhibitory properties of silibinin. Brush‐shaped nanoparticles can carry multiple drugs simultaneously for the codelivery of silibinin and other anticancer drugs [76]. This suggests strategy for improving the design of current first‐line combination therapies for NSCLC.

## Author Contributions

**Xiaocen Wang:** methodology, conceptualization, investigation, writing – original draft, writing – review and editing, resources, data curation, supervision.

## Ethics Statement

The author has nothing to report.

## Conflicts of Interest

The author declares no conflicts of interest.

## Data Availability

All raw data and code are available upon request.
